# The (Un)Attractiveness of Dark Triad Personalities: Assessing Fictitious Characters for Short‐ and Long‐Term Relationships

**DOI:** 10.1111/jopy.12994

**Published:** 2024-11-18

**Authors:** Yavor Dragostinov, Tom Booth

**Affiliations:** ^1^ Department of Psychology University of Edinburgh Edinburgh UK; ^2^ School of Social Sciences Heriot‐Watt University Edinburgh UK

**Keywords:** attractiveness, Dark Triad, mixed models, personality, relationships

## Abstract

**Objective:**

The current study assessed how individuals evaluate potential romantic partners who display either low, medium, or high levels of DT traits for short‐term (STR) and long‐term (LTR) relationships.

**Methods:**

Nine fictitious persons in the form of vignettes (description of behavior and facial image) were presented to every participant. The sex of the fictitious persons was determined by sexual orientation of each participant, while the displayed faces were selected from an existing image bank and matched for physical attractiveness. Study 1 (*n* = 475) used a fixed composition for face and trait description, while the composition for Study 2 (*n* = 794) was randomized. Mixed‐effects modeling was implemented for both studies.

**Results:**

Study 1 demonstrated people with a male preference (mostly women) perceived medium levels of the three traits as the most attractive STR. For Study 2, both men and women found the low levels the most attractive for both STRs and LTRs.

**Conclusions:**

Findings from Study 1 were mostly consistent across previous DT attractiveness literature, while findings from Study 2 contradicted them. This could suggest that the concept of DT is not as attractive even for STRs unless it is accompanied by physical attractiveness.

The Dark Triad (DT) consists of three personality traits—narcissism, Machiavellianism, and subclinical psychopathy (Paulhus and Williams, 2002). DT traits are not interchangeable although there are phenotypical behaviors which are consistent across all three traits such as egocentricity, manipulation, and exploitation (Rauthmann and Will 2011). As such, DT traits show consistent patterns of positive correlations, regardless of the measurement being used (Furnham, Richards, and Paulhus 2013). Moreover, evidence demonstrates a positive association between DT and variables such as forcefulness, antisocial behavior, disinhibition (Jones and Paulhus 2011), interpersonal antagonism (Furnham, Richards, and Paulhus 2013) as well as a strong and consistently negative association with the trait agreeableness (Jakobwitz and Egan 2006; Paulhus and Williams 2002). Such behaviors are believed to be fitness‐increasing, thus they are expected to be adaptive when it comes to mate choice (Buss and Duntley 2008). In fact, there is a large body of research arguing that DT is advantageous to certain men, especially in terms of mating (Paulhus and Williams 2002; Foster, Campbell, and Twenge 2003; Jonason, Li, and Buss 2010). The existing research has focused heavily on female mate choice, as only a limited work has focused on how men perceive romantic partners who have DT tendencies. Moreover, the research has typically looked at high versus low levels of DT, with little acknowledgement of the fact traits usually measure a continuum. This study will attempt to fill in those gaps by considering both male and female mate choice, and considering targets with average (medium) levels of DT traits.

## 1 Perceived Attractiveness of the Dark Triad

The DT has been widely researched in the context of mate choice and sexual behavior. One area that has received comparatively little attention is the perceived attractiveness of DT traits. A variety of methods have been used to evaluate the perceived attractiveness of DT individuals, of which assessment of computerized faces is the most common (e.g., Lyons and Simeonov 2016; Brewer et al. 2018; Marcinkowska, Lyons, and Helle 2016). In each of these studies, images of low and high DT individuals were taken from Holtzman (2011) set of digitally manipulated faces. So far, findings appear to be inconsistent.

Narcissism has been reported to be perceived as a desirable trait for both short‐term (STR) and long‐term (LTR) relationships (Lyons and Blanchard 2016, Study 2), while Machiavellianism has been associated with desirability for STR (Lyons and Simeonov 2016). However, this effect was only observed in the condition where the vignettes were describing people from a wealthy background. Moreover, facial features of all the DT traits were perceived as significantly attractive (Marcinkowska, Lyons, and Helle 2016). Despite having identical designs, the remaining studies report contradictory findings. Women reportedly disliked DT faces for both STR and LTR (Lyons and Blanchard 2016, Study 1; Lyons et al. 2015; Brewer et al. 2018). There are a number of factors that could explain these inconsistent findings.

First, it has been documented that women's preference for male faces changes based on individual differences (DeBruine et al. 2006) and sociosexual orientation (Waynforth, Delwadia, and Camm 2005, cited from Lyons et al. (2015)). Second, there is an element of DT that cannot be captured from simply evaluating facial morphs—people's behavior. For example, Back and associates (Back, Schmukle, and Egloff 2010, Study 1) conducted an experiment to evaluate the popularity of narcissists at first sight. Participants were given a randomly assigned seat number in a student classroom. They were requested to individually step forward to a marked spot and briefly introduce themselves. The self‐introductions were videotaped and ranged from 4 to 21 s. At the end of each introduction, the participants were evaluated by other freshmen. This continued until every participant introduced themselves. Finally, the participants were given a narcissism questionnaire and a popularity measure to complete at home. While the study had a limited sample size (*n* = 73), each participant rated all other participants on two rating scales. This resulted in 73 × 72 × 2 = 10,512 ratings. Overall, the correlation between individuals perceived as narcissistic and their popularity was at 0.21.

Findings such as that of Back et al. (2010, Study 1) indicate that to fully explore the association between DT and attractiveness ratings, it may be beneficial to consider not only physical attractiveness, but also aspects of behavior. One possible approach to addressing this issue is to adopt hypothetical behavioral scenarios. Studies which have applied this methodology have relied on fictitiously filled in questionnaires (Dufner et al. 2013, Study 1) or vignettes which describe either fictitious (Carter, Campbell, and Muncer 2014; Rauthmann and Kolar 2013; Aitken, Lyons, and Jonason 2013; Lyons and Simeonov 2016) or actual persons (Jonason, Lyons, and Blanchard 2015) of the opposite sex who score high or low on a DT measurement (e.g., Dirty Dozen, Jonason and Webster 2010).

Carter and associates (Carter, Campbell, and Muncer 2014) demonstrated that narcissism (*t* = 8.40, *p* < 0.001, *d* = 1.33), Machiavellianism (*t* = 10.91, *p* < 0.001, *d* = 1.73) and psychopathy (*t* = 7.06, *p* < 0.001, *d* = 1.81) were all significantly preferred for STR compared to the controls. Aitken, Lyons, and Jonason (2013) evaluated women's perception of men with high and low levels of Machiavellianism. When contemplating STR, women preferred high level Machiavellian vignettes (*t* = −2.60, *p* < 0.05, *d* = 0.20). In contrast, low level vignettes were significantly preferred for LTR (*t* = 4.14, *p* < 0.01, *d* = 0.30). Jonason, Lyons, and Blanchard (2015) evaluated the desirability of all DT traits using dating adverts. For STR, individuals with high levels of Machiavellianism were preferred over low levels by men (*F* = 19.57, *p* < 0.001, *d* = 0.28) and women (*F* = 7.70, *p* < 0.01, *d* = 0.12). High level psychopathy adverts were found as more attractive for STR compared to low levels by men (*F* = 21.90, *p* < 0.001, *d* = 0.24) and women (*F* = 7.50, *p* < 0.01, *d* = 0.12). For narcissism, adverts showing high levels of narcissism were considered more attractive than low levels for STR by women (*F* = 6.18, *p* < 0.01, *d* = 0.14). The opposite was demonstrated for LTR. Low levels of Machiavellianism were significantly preferred over high levels by men (*F* = 179.46, *p* < 0.001, *d* = 0.79) and women (*F* = 224.76, *p* < 0.01, *d* = 0.80). This was the case for psychopathy by men (*F* = 152.00, *p* < 0.01, *d* = 0.68) and women (*F* = 177.80, *p* < 0.01, *d* = 0.77), as well as for narcissism by men (*F* = 44.01, *p* < 0.01, *d* = 0.49) and women (*F* = 19.92, *p* < 0.01, *d* = 0.18). To summarize, there are findings suggesting women find all three high‐level DT traits attractive for STR compared to lower levels. Evidence regarding which of the three traits is perceived the most attractive is mixed. For LTR, low‐level DT traits are considered the most attractive.

There are some further limitations that need to be addressed, such as stimulus. Some studies (Rauthmann and Kolar 2013; Carter, Campbell, and Muncer 2014; Dufner et al. 2013) evaluated the attractiveness of the DT using only vignettes or supposedly filled in questionnaires with no images attached to the fictitious persons. This means participants most likely used their imagination to picture the physical appearance of each fictitious person. This is particularly problematic as it can generate answers inspired by unrealistic ideals or past romantic experiences. Selecting romantic partners based on photos and self‐reported behavioral descriptions is mirrored in real‐life scenarios. Dating apps like Tinder, Hinge, Feeld, and others have revolutionized the landscape in which people pursue romantic connections (LeFebvre 2018). For instance, the Hinge platform offers varied prompts, such as “dating me is like…” and “facts about me that surprise people…,” allowing users to respond in an open‐ended fashion. This approach can be seen as a blend of the facial and behavioral study designs discussed earlier. Chopik and Johnson (2021) emphasized that with the growing prominence of such dating apps, “the field of relationship science should invest more in understanding attraction, dating, and relationship maintenance” (10). Our objective is for this study to further that understanding.

A number of studies have presented faces to the subjects, however most of them (Aitken, Lyons, and Jonason 2013; Jonason, Lyons, and Blanchard 2015) did not rate physical attractiveness of the faces prior to the experiment. This means there is no way to know how much physical attractiveness influenced the selection of DT individuals. Only one study (Dufner et al. 2013, Study 2) rated attractiveness. Dufner and associates analyzed narcissism and self‐esteem from data of heterosexual participants for whom peer ratings were available (*n* = 152, 75.66% female). Each subject invited one close friend to rate them on appeal, friend appeal, physical attractiveness and social boldness. Therefore, despite being able to control for these features of the individual, each participant was only rated by one close friend. Moreover, 82.89% of the participants chose a rater of the same sex, while 65.13% of the raters knew the person they were assessing. These limitations were acknowledged by the authors.

Another notable ongoing limitation of the DT literature is the overwhelming focus on female mate choice. Out of the reviewed studies (both facial morphs and behavioral), only two did not use a strictly female sample (Rauthmann and Kolar 2013; Dufner et al. 2013). While the personality literature is very consistent—men appear to score higher than women on DT personality traits (Allsopp, Eysenck, and Eysenck 1991; Mealey 1995; Jonason et al. 2009), it is not clear why the majority of the studies on perception of DT have neglected male mate choice. Moreover, we could not find studies assessing how non‐heterosexual participants perceive DT personalities in romantic settings. However, regarding short‐term relationships, a large cross‐national study by Lippa (2007) found very similar mean levels of sex drive among heterosexual, homosexual, and bisexual participants.

Lastly, almost all of the DT perception studies have focused on comparing low and high‐level DT levels. Only one study (Dufner et al. 2013, Study 1) included a medium level. Having a medium level would present participants with a subcomponent option. This is something that has been missing in the literature and has been recommended by previous work (Carter, Campbell, and Muncer 2014). Furthermore, subcomponents of the DT are more common in real‐world settings in comparison to low or high levels (Jonason, Icho, and Ireland 2016; Carter, Campbell, and Muncer 2014).

## 2 The Current Work

The present study extends previous work by exploring the attractiveness of DT traits using vignettes combined with images from the Chicago Face Dataset (Ma, Correll, and Wittenbrink 2015) that have been selected to control for attractiveness. In an initial pilot study, vignettes were designed to assess low, medium, and high levels of each DT trait.

Next, we assess the preferences of both male and female participants for STR and LTR using these vignettes, where the sex of the rated targets is determined by the self‐reported sexual orientation of the participants. In Study 1, images were presented in a fixed order, whereas in Study 2, presentation was randomized.

Based on the previous findings we tested the following hypotheses:

Hypothesis 1 *Those with a male sexual preference will perceive the high‐level DT character as the most attractive for STR*.

Hypothesis 2 *The low‐level DT character will be perceived as the most attractive for LTR by those with a male and those with a female sexual preference*.

### 2.1 Pilot Study: Vignette Development

Similar to previous works (Rauthmann and Kolar 2013; Carter, Campbell, and Muncer 2014; Aitken, Lyons, and Jonason 2013), this study adopted a vignette approach. Low and high‐level vignettes were taken from the Dirty Dozen (Jonason and Webster 2010). We also created medium‐level vignettes, as the primary focus was on frequency (sometimes, tends to, admits he/she has). As we developed the medium descriptors, we conducted a pilot study to understand whether participants could identify the trait and level of each vignette.

### 2.2 Participants

Participants (*n* = 29) were recruited via opportunity sample of postgraduate psychology students from the University of Edinburgh (*n* = 12), and social media platforms such as Facebook (*n* = 8) and Instagram (*n* = 9).

### 2.3 Materials and Procedure

At the start of the pilot study, participants were instructed that they would read brief descriptions in the forms of vignettes which describe either low, medium or high‐level DT trait (narcissism, Machiavellianism, and psychopathy). Brief definitions and examples of all DT traits were also presented to the participants. After reading each vignette, participants had to select which trait the vignette represented (“Which trait is this referring to?”) as well as which level (“Which level of the selected trait does the vignette describe?”). The initial and revised vignettes can be found in the Supporting Information.

### 2.4 Results

Percentage of accurate identification of level, trait and the combination of trait and level were evaluated (see Table 1). Arbitrarily, we took the accuracy of > 80% to be indicative of good levels of recognition. The highest percentage of trait recognition for low‐level DT was 0.55, which did not approach our accuracy threshold. Level recognition was satisfactory. Medium‐level DT reported better scores for trait recognition than the low level, but not for level recognition. High‐level DT reported the highest percentage scores for trait recognition. Narcissism and Machiavellianism reported good percentages for level recognition, however psychopathy reported an extremely low accuracy percentage.

**TABLE 1:** Percentages of correct interpretations of trait and level of the respective vignette.

	Narcissism	Machiavellianism	Psychopathy
*Low*			
Trait	0.41	0.51	0.55
Level	0.93	0.86	0.93
Combined	0.41	0.37	0.51
*Medium*			
Trait	0.86	0.79	0.62
Level	0.62	0.55	0.72
Combined	0.55	0.41	0.51
*High*			
Trait	1	0.86	0.89
Level	0.79	0.86	0.17
Combined	0.79	0.79	0.17

Overall, the accuracy percentages were unsatisfactory. As such, we conducted a revision exercise of all vignettes via consultation with subject matter experts (SME).

## 3 Subject Matter Experts Consultation

Five subject matter experts (SME) were contacted and asked to review the pilot study results and the overall structure of the vignettes. All researchers have produced substantial work in the field of personality and individual differences (for the names of the researchers who were contacted, please see “Acknowledgements”).

For narcissism, the majority of experts offered positive feedback. It was noted by multiple SME that the poor trait recognition regarding the low narcissism vignette may be due to uncertainty over “anti‐narcissism.” Subjects do recognize that the low vignette is describing a nice person, but they are unsure how nice they are.

Most of the SME agreed that the biggest concern with the vignettes was the discrimination between Machiavellianism and psychopathy. Some experts suggested most of the psychopathy vignettes were describing hallmarks of Machiavellianism and vice‐versa.

Another concern was the overall context of Machiavellianism and psychopathy vignettes. One expert questioned why a Machiavellian would have trouble understanding other people's feelings, as taking advantage of one's feelings is considered a reoccurring tendency of a prototypical Machiavellian. In terms of the psychopathy vignette, experts noted that it would be more beneficial if the vignettes focused on lack of empathy rather than frustration intolerance.

The SME agreed that the extremely low score regarding level recognition for high psychopathy was likely a result of poor wording in the vignette. It was suggested that “he is not too concerned about the morality of his actions…” needs to be changed to “he is not at all concerned of the morality of his actions….”

The experts advised that the vignettes are too long, especially at the medium level. Multiple experts suggested that the medium vignettes are putting two ideas in the same sentence (“He does A, but not B”), rather than sticking to one tendency per sentence (“He sometimes does A, but not always”; “You can seem him doing B, but that is not too often”). Based on this feedback and recommendations, all vignettes were updated (see Supporting Information). In summary, the core changes included:

1. Reducing the length of the vignettes.
2. Simplifying each sentence to only reflect a single behavior.
3. Correcting the inconsistencies regarding the distinction between Machiavellianism and psychopathy.

The updated vignettes are free to use and were used in the subsequent studies.

## 4 Study 1

### 4.1 Participants

Participants (*n* = 475, *M*_age_ = 27.00, *SD* = 7.12^1^; 344 females, *M*_age_ = 25.62, *SD* = 4.77; 160 males, *M*_age_ = 28.68, *SD* = 9.12; 5 non‐binary, *M*_age_ = 30.50, *SD* = 6.86) were recruited online using social platforms such as Facebook and Instagram, as well as the survey sharing site SurveyCircle. 320 of the participants reported a male sexual preference, 156 reported a female preference, while 32 participants indicated a preference for both sexes. As can be seen in Table 2, a majority of the participants were heterosexual, reporting an opposite preference to self‐reported gender. Subjects did not receive financial compensation for completing the survey.

**TABLE 2:** Gender and sexual preference demographics for Study 1.

Preference	Gender			Total
	Male	Female	Non‐binary	
Male	24	264	2	290
Female	129	22	2	153
Both	4	27	1	32
	157	313	5	475

We used G*Power to determine the likelihood of detecting a significant effect with a sample size of 475, an alpha level of 0.05, and a power of 0.90. The results indicated that the study has a 90% chance of detecting an effect size of *d* = 0.21 (Cohen 1988). This analysis is not a post hoc power analysis but rather an a priori determination of the study's ability to detect a meaningful effect (Lakens 2022).

### 4.2 Materials and Procedure

A total of nine different fictitious persons were presented to participants (3 DT traits × 3 levels). Each “person” consisted of a vignette describing a low, medium or high DT personality and an image of a face. The faces that were attached to each vignette were 2D colorful images from the neck up with a neutral facial expression and all of them were of Caucasian ethnicity. The images were taken from the Chicago Face Dataset (Ma, Correll, and Wittenbrink 2015), which had attractiveness ratings for each face that ranged from 0 to 5. Only faces which were rated as either 3 or 4 were selected. All faces were within the same age range (mid‐twenties). The final selection of the faces was based on the authors' judgment.

Participants had to rate the nine fictitious persons on attractiveness for a short‐term relationship (“How much would you like this person for a short‐term sexual affair?”) and long‐term relationship (“How much would you like this person for a committed long‐term relationship?”) on a 5‐point Likert scale (1–5). Those who stated a male sexual preference saw nine male faces and those who stated a female sexual preference saw nine female faces. Individuals who stated a preference for both sexes saw a mix—four female faces and five male faces. These faces were a subset of the faces shown to the groups with either a male‐only or female‐only preference. The image‐vignette couples followed a fixed order. To see which specific face was paired with each vignette, please refer to the “Materials” folder in the OSF repository.

#### 4.2.1 Analysis Strategy

Models were fitted using the package lmer4 version 1.1‐21 (Bates et al. 2015) in R version 1.2.1335. The models were estimated using restricted maximum likelihood (REML) and nlopwrap optimizer. Models were fit to each of the DT traits and relationship length separately. We elected to stratify by relationship length to avoid using three‐way interactions due to concerns about interpretation complexity (Aiken, West, and Reno 1991) and power.

Initial models were estimated with random intercepts for participant and face (see Judd et al. 2012) to calculate the intraclass correlation coefficient (ICC). For STR models, ICCs were 0.39, 0.31, and 0.40 for Narcissism, Machiavellianism, and Psychopathy, respectively. For LTR models, ICCs were 0.51, 0.37, and 0.50 for Narcissism, Machiavellianism, and Psychopathy, respectively.

Sexual preference was dummy coded using male as the reference group, while DT level was coded as an ordered variable using low as the reference group. To allow the testing of the ordered effects of the level of DT, forward difference coding was implemented resulting in two coefficients for level; DT Level 1 represents the average difference in attractiveness between the low and medium level, with positive values indicating low has the higher mean level. DT Level 2 describes the average difference between the medium and high level, with positive values indicating that medium has the higher mean level. For sexual preference, “Sexual Preference: Female” represents the average difference in attractiveness perception in people with a male and female preference, while “Sexual Preference: Both” represents the average difference in perception of attractiveness in those with a female and “both” preferences.

Three primary models of interests were introduced:

- Model 1 (M1) predicted attractiveness from sexual preference with participant and face retained as random effects.
- Model 2 (M2) predicted attractiveness from sexual preference and the level of the DT trait with participant and face retained as random effects.
- Model 3 (M3) predicted attractiveness from the interaction between sexual preference and the level of the DT trait with participant and face retained as random effects.

Models were compared based on a range of model fit indices to identify the best model. Specifically, model fit was estimated using the Akaike information criteria (AIC) and Bayesian information criteria (BIC). Whenever AIC and BIC indicated different models had optimal fit, decisions were based on the BIC as it has a stricter penalty for model parsimony and will thus favor the simpler model (Shoemaker 2021).

#### 4.2.2 Sensitivity Analysis

To ensure that there were not important differences in relationship preference/behavior between heterosexual, homosexual, and bisexual participants from our samples, a sensitivity analysis was conducted using the models from both studies. Each model was run twice—once with all of the participants and once using only the heterosexual sample. The results showed minimal differences, as the differences of the effects between the two samples were within the 95% confidence intervals (see Figure S1). Due to the lower number of participants identifying as having a same sex (*n* = 46) or mixed sex preference (*n* = 32) were low, we were unable to run a stratified analysis with just this sample.

### 4.3 Results

Model fit results for Study 1 and Study 2 are shown in Table 3. In Study 1, the simplest model (M1) provided the best fit for each of DT traits. The results of the sensitivity analysis across both studies showed minimal differences in the parameter estimates from the preferred models, based on overlapping 95% confidence intervals. As such, for the rest of the manuscript, we report results from the total sample. The sensitivity analysis and the code for it can be found in the Supporting Information.

**TABLE 3:** Model fit indices for mixed models in Study 1.

		AIC	BIC	LRT
Study 1: STR	**M1: Narcissism**	**4186.10**	**4217.67**	**−2087.05**
	M2: Narcissism	4189.59	4231.68	−2086.79
	M3: Narcissism	4194.44	4257.58	−2085.22
	**M1: Machiavellianism**	**4108.77**	**4140.35**	**−2048.39**
	M2: Machiavellianism	4112.92	4155.02	−2048.46
	M3: Machiavellianism	4116.57	4179.71	−2046.29
	**M1: Psychopathy**	**4082.10**	**4113.67**	**−2035.05**
	M2: Psychopathy	4083.65	4125.74	−2033.82
	M3: Psychopathy	4086.31	4149.46	−2031.16
Study 1: LTR	**M1: Narcissism**	**3926.71**	**3958.28**	**−1957.36**
	M2: Narcissism	3921.63	3963.72	−1952.81
	M3: Narcissism	3927.15	3990.29	−1951.57
	**M1: Machiavellianism**	**3973.15**	**4004.72**	**−1980.57**
	M2: Machiavellianism	3970.82	4012.91	−1977.41
	M3: Machiavellianism	3975.60	4038.74	−1975.80
	**M1: Psychopathy**	**3923.21**	**3954.78**	**−1955.60**
	M2: Psychopathy	3916.86	3958.96	−1950.43
	M3: Psychopathy	3922.38	3985.52	−1949.19

*Note:* Bold indicates the best fit model according to our criteria. Model 1 (M1) predicted attractiveness from sexual preference with participant and face retained as random effects. Model 2 (M2) predicted attractiveness from sexual preference and the level of the DT trait with participant and face retained as random effects. Model 3 (M3) predicted attractiveness from the interaction between sexual preference and the level of the DT trait with participant and face retained as random effects.

#### 4.3.1 Short‐Term Attractiveness

Results for the preferred models of STR are shown in Table 4. For both Machiavellianism and Narcissism, the random effects for person (ID) are larger than the random effect of face stimuli. Interestingly, in the model for Psychopathy, the face stimuli random effect is the largest, suggesting a greater influence of stimuli for Psychopathy.

**TABLE 4:** Results predicting attractiveness for STR from DT traits (Study 1).

Predictors	Narcissism			Machiavellianism			Psychopathy		
	Estimates	*SE*	*p*	Estimates	*SE*	*p*	Estimates	*SE*	*p*
Intercept	1.93	0.15	< 0.001	2.02	0.14	< 0.001	1.99	0.21	< 0.001
Sexual preference: Female	0.87	0.17	< 0.001	−0.17	0.17	0.324	0.34	0.19	0.073
Sexual preference: Both	0.55	0.19	0.003	−0.01	0.15	0.933	0.22	0.15	0.145
*Random effects*									
*σ* ^2^	0.78			0.81			0.77		
*τ*_00 ID_	0.42			0.28			0.30		
*τ*_00 face_	0.09			0.08			0.21		

*Note: N*_ID_ = 475; Observations = 1425.

For Narcissism, we see a significant difference in the perceived attractiveness ratings of those with female preference over those with male preferences (*β* = 0.87, *SE* = 0.17, *p* < 0.001). Similarly, those with a “both” preference reported significantly higher attractiveness ratings than those with male preference (*β* = 0.55, *SE* = 0.17, *p* < 0.003). For both Machiavellianism and Psychopathy, despite the preference model showing optimal fit, none of the differences between the groups reached significance.

The preferred models did not contain the interaction between preference and DT trait level; thus Hypothesis 1 was not supported.

The mean attractiveness (i.e., 1–5) ratings for STR for those with a male sexual preference (i.e., mostly women) ranged from 1.12 to 2.13 (*SD* range: 0.70–1.13; see Table S1). The mean attractiveness ratings (see Table S2) of those with a female sexual preference (i.e., mostly men) ranged from 1.99 to 3.18 (*SD* range: 1.05–1.26). The normative ratings of each specific face can be found in the OSF repository.

#### 4.3.2 Long‐Term Attractiveness

Results for LTR are shown in Table 5. In contrast to models of STR, random effects of face stimuli are larger than the random effects associated with ID for each of the DT traits. This indicates that for LTR, variance in ratings may be more influenced by the facial stimuli.

**TABLE 5:** Results predicting attractiveness for LTR from DT traits (Study 1).

Predictors	Narcissism			Machiavellianism			Psychopathy		
	Estimates	*SE*	*p*	Estimates	*SE*	*p*	Estimates	*SE*	*p*
Intercept	1.83	0.30	< 0.001	1.97	0.24	< 0.001	1.72	0.04	< 0.001
Sexual preference: Female	0.46	0.19	0.016	−0.31	0.19	0.109	0.51	0.06	< 0.001
Sexual preference: Both	0.41	0.18	0.021	0.08	0.14	0.580	0.20	0.12	0.085
*Random effects*									
*σ* ^2^	0.70			0.78			0.76		
*τ*_00 ID_	0.25			0.17			0.16		
*τ*_00 face_	0.49			0.28			0.60		

*Note: N*_ID_ = 475; Observations = 1425.

For both Narcissism (*β* = 0.46, *SE* = 0.19, *p* = 0.02) and Psychopathy (*β* = 0.51, *SE* = 0.06, *p* < 0.001), those with a female preference reported, on average, higher attractiveness ratings than those with a male preference. For Narcissism, as with STR, those with "both" preference also reported higher average perceived attractiveness than those with male preference (*β* = 0.41, *SE* = 0.18, *p* < 0.05). For Machiavellianism, no differences between groups were statistically significant.

The preferred models did not contain the interaction between preference and DT trait level, thus Hypothesis 2 was not supported.

The mean attractiveness ratings for LTR for those with a male sexual preference ranged from 1.12 to 2.37 (*SD* range: 0.39–1.14). The mean attractiveness ratings of those with a female sexual preference ranged from 1.33 to 3.39 (*SD* range: 0.74–1.33).

#### 4.3.3 Interaction Effects of Dark Triad Levels and Relationship Type

Additionally, a linear mixed effects model was run to examine the effect of DT levels (low, medium, and high) and relationship type (short‐term and long‐term) on attractiveness ratings. The model also included random intercepts for face and ID.

The results showed a significant interaction between DT levels and relationship type (Table 6). Specifically, the interaction between higher DT traits and short‐term relationships was significant, *β* = −0.66, *SE* = 0.05, *t*(8054.05) = −14.24, *p* < 0.001, indicating that the positive effect of medium and high level DT traits on attractiveness was reduced in short‐term relationships. Similarly, the interaction between high DT traits and short‐term relationships was also significant, *β* = −0.27, *SE* = 0.05, *t*(8054.05) = −5.95, *p* < 0.001. This suggests that while higher DT levels generally increase attractiveness, this effect is less pronounced in short‐term relationships (see Figure S3).

**TABLE 6:** Interaction between Dark Triad levels and relationship type (Study 1).

Predictors	Attractiveness		
	Estimates	*SE*	*p*
Intercept	1.96	0.09	< 0.001
Level: 1	0.78	0.20	< 0.001
Level: 2	0.57	0.20	0.005
Relationship: Short	0.17	0.02	< 0.001
Level: 1 × Relationship: Short	−0.66	0.05	< 0.001
Level: 2 × Relationship: Short	−0.27	0.05	< 0.001
*Random effects*			
	*σ* ^2^	*τ*_00 ID_	*τ*_00 face_
	0.76	0.28	0.12

*Note: N*_ID_ = 475; Observations = 8550.

For long‐term relationships, higher DT levels generally increased attractiveness more compared to short‐term relationships, supporting Hypothesis 2 that lower DT levels are perceived as more attractive for long‐term relationships.

#### 4.3.4 Interaction Effects of Sexual Preference and Relationship Type

Another linear mixed‐effects model was conducted to investigate the interaction between sexual preference (male, female, and "both") and relationship type on attractiveness ratings. This model also included random intercepts for face and ID.

Results showed a significant interaction between female sexual preference and short‐term relationships, *β* = 0.20, *SE* = 0.04, *t*(8055.03) = 4.79, *p* < 0.001, indicating that female preference significantly increased attractiveness in short‐term relationships (Table 7). The interaction between preference for both sexes and short‐term relationships was not significant, *β* = −0.02, *SE* = 0.08, *t*(8055.03) = −0.24, *p* = 0.81. Notably, for individuals with a male preference, there was no significant interaction with relationship type, suggesting that the effect of relationship type on attractiveness was stable regardless of whether the relationship was short‐term or long‐term (see Figure S4).

**TABLE 7:** Interaction between sexual preference and relationship type (Study 1).

Predictors	Attractiveness		
	Estimates	*SE*	*p*
Intercept	1.87	0.12	< 0.001
Sexual preference: Female	0.15	0.10	0.12
Sexual preference: Both	0.21	0.12	0.10
Relationship: Short	0.11	0.02	< 0.001
Sexual preference: Female × Relationship: Short	0.20	0.04	< 0.001
Sexual preference: Both × Relationship: Short	−0.02	0.08	0.81
*Random effects*			
	*σ* ^2^	*τ*_00 ID_	*τ*_00 face_
	0.80	0.28	0.22

*Note: N*_ID_ = 475; Observations = 8550.

### 4.4 Discussion

We found no evidence to support either hypothesis as the models containing the level‐preference interaction were not optimal based on model fit criteria. Within the models estimated, consistent differences by preference were shown in the average attractiveness ratings for Narcissism, with female preference reporting the highest levels of perceived attractiveness across both STR and LTR. No effects of preference were found for Machiavellianism. For LTR, we found similar patterns as seen for Narcissism and Psychopathy.

For individuals with a male preference, there was no significant interaction with relationship type, suggesting that the effect of relationship type on attractiveness was stable regardless of whether the relationship was short‐term or long‐term. This does not support Hypothesis 1, as higher DT levels did not significantly increase attractiveness for those with a male preference in short‐term relationships. For Hypothesis 2, the results suggest that attractiveness ratings were more stable for male preferences across relationship types.

## 5 Study 2

### 5.1 Participants

Participants (*n* = 794, *M*_age_ = 27.61, *SD* = 9.11; 345 females, *M*_age_ = 29.37, *SD* = 9.60; 442 males, *M*_age_ = 26.33, *SD* = 8.54; 8 non‐binary, *M*_age_ = 21.75, *SD* = 2.31) were recruited using the paid platform Prolific. Only participants who were fluent in English were selected for the study. 312 of the participants reported a male sexual preference, 429 reported a female preference and 61 subjects reported a preference for both sexes (Table 8). As with Study 1, Table 6 shows that a majority of the sample were heterosexual, reporting an opposite preference to self‐reported gender. For this study, the rate was £5.68/h, with a median completion time of 3 min and 42 s. As per the demographics provided by Prolific, 382 of the subjects were enrolled students at the time of completing the survey.

**TABLE 8:** Gender and sexual preference demographics for Study 2.

Preference	Gender			Total
	Male	Female	Non‐binary	
Male	34	275	0	309
Female	392	32	1	425
Both	14	39	7	60
	440	346	8	794

We used G*Power to determine the likelihood of detecting a significant effect with a sample size of 794, an alpha level of 0.05, and a power of 0.90. The results indicated that the study has a 90% chance of detecting an effect size of *d* = 0.16 (Cohen 1988). Like Study 1, this analysis is not a post hoc power analysis but rather an a priori determination of the study's ability to detect a meaningful effect (Lakens 2022).

### 5.2 Design

The faces from Study 1 were used for the design of Study 2 as well. The number of fictitious persons being rated, and the structure of the blocks mirrored those from Study 1. The key difference was that the order of the faces being displayed was randomized. This means the physical attractiveness of each personality was represented by a different face for each participant. Those who stated a male sexual preference saw nine male faces and those who stated a female sexual preference saw nine female faces. Individuals who stated a preference for both sexes saw a mix (four female faces and five male faces).

#### 5.2.1 Analysis Strategy

The analysis strategy followed the same steps as in Study 1. We included random intercepts for both participant and face stimuli. DT level was again coded using forward difference coding, with preference dummy coded uses male as the reference. We again fit the same series of three models, and assessed the best model based on model fit comparisons (Table 9). For STR, the ICC was at 0.30, 0.26, and 0.27 for Narcissism, Machiavellianism, and Psychopathy, respectively.

**TABLE 9:** Model fit indices for Study 2.

		AIC	BIC	LRT
Study 2: STR	M1: Narcissism	7218.59	7253.24	−3603.30
	**M2: Narcissism**	**7032.92**	**7079.12**	**−3508.46**
	M3: Narcissism	7032.10	7101.41	−3504.05
	M1: Machiavellianism	7320.08	7354.74	−3654.04
	**M2: Machiavellianism**	**7136.52**	**7182.73**	**−3560.26**
	M3: Machiavellianism	7141.71	7211.02	−3558.86
	M1: Psychopathy	7415.39	7450.04	−3701.70
	M2: Psychopathy	7245.26	7291.47	−3614.63
	**M3: Psychopathy**	**7220.53**	**7289.84**	**−3598.26**
Study 2: LTR	M1: Narcissism	7684.16	7718.82	−3836.08
	**M2: Narcissism**	**6654.86**	**6701.07**	**−3319.43**
	M3: Narcissism	6656.59	6725.90	−3316.30
	M1: Machiavellianism	7633.07	7667.73	−3810.54
	**M2: Machiavellianism**	**6744.97**	**6791.17**	**−3364.48**
	M3: Machiavellianism	6759.45	6828.75	−3367.72
	M1: Psychopathy	7686.03	7720.68	−3837.01
	**M2: Psychopathy**	**6656.77**	**6702.97**	**−3320.38**
	M3: Psychopathy	6649.09	6718.40	−3312.54

*Note:* Bold indicates the best fit model according to our criteria. Model 1 (M1) predicted attractiveness from sexual preference with participant and face retained as random effects. Model 2 (M2) predicted attractiveness from sexual preference and the level of the DT trait with participant and face retained as random effects. Model 3 (M3) predicted attractiveness from the interaction between sexual preference and the level of the DT trait with participant and face retained as random effects.

#### 5.2.2 Sensitivity Analysis

Similar to Study 1, we conducted a sensitivity analysis where were limited the sample to only participants identifying an opposite sex preference. The results again showed minimal differences, as the differences of the effects between the two samples were within the 95% confidence intervals (see Figure S2).

### 5.3 Results

As can be seen in Table 9, M2 which includes both DT trait level and preference as main effects but no interaction, was the preferred model for STR Narcissism, Machiavellianism and all models for LTR. For STR psychopathy, the interaction model (i.e., M3) provided the best fit. In both the STR and LTR models, the random effect of face stimuli was near zero.

#### 5.3.1 Short‐Term Attractiveness

For Narcissism, we see the same effects of preference as demonstrated in Study 1. Those with female preference (*β* = 0.71, *SE* = 0.07, *p* < 0.001) and mixed preference (*β* = 0.43, *SE* = 0.11, *p* < 0.001), show a significantly higher mean attractiveness rating than those with male preference. For DT level, low‐level was perceived as more attractive than the medium‐level (*β* = 0.47, *SE* = 0.05, *p* < 0.001), while the medium‐level was perceived as more attractive than the high‐level (*β* = 0.17, *SE* = 0.05, *p* < 0.001).

A highly similar pattern is shown for Machiavellianism. Those reporting female preference show significantly higher average attractiveness ratings than those with male preference (*β* = 0.70, *SE* = 0.07, *p* < 0.001). For DT level low‐level was perceived as more attractive overall than the medium (*β* = 0.40, *SE* = 0.05, *p* < 0.001), while the medium was perceived as more attractive than the high‐level (*β* = 0.30, *SE* = 0.05, *p* < 0.001).

**FIGURE 1:**
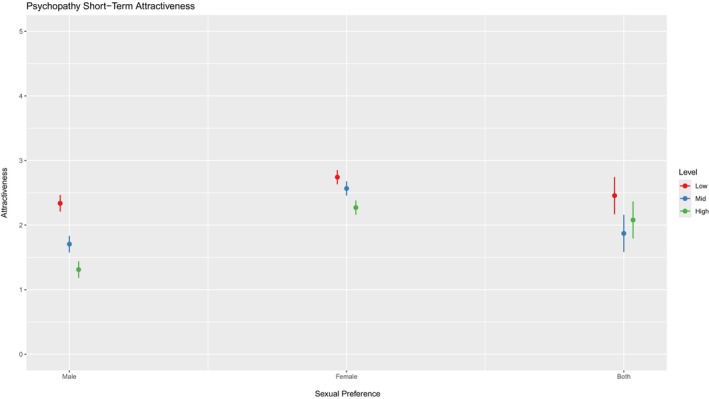
Plot of the interaction model.

For Psychopathy, the interaction between the average difference of attractiveness in male and female preference for low and medium level psychopaths was negative and significant (*β* = −0.46, *SE* = 0.10, *p* < 0.001). This suggests that individuals with a female preference found the low‐level as more attractive compared to those with a male preference (see Figure 1). Individuals with a female preference also found the medium and high levels of psychopathy slightly more attractive in comparison to those with a male preference (*β* = −0.10, *SE* = 0.10, *p* = 0.33). This shows that attractiveness of men did not increase for people with a male preference (see Table 10).

**TABLE 10:** Results predicting attractiveness for STR from DT traits (Study 2).

Predictors	Narcissism			Machiavellianism			Psychopathy		
	Estimates	*SE*	*p*	Estimates	*SE*	*p*	Estimates	*SE*	*p*
Intercept	1.86	0.05	< 0.001	1.84	0.05	< 0.001	1.78	0.05	< 0.001
Sexual preference: Female	0.71	0.07	< 0.001	0.70	0.07	< 0.001	0.74	0.06	< 0.001
Sexual preference: Both	0.43	0.11	< 0.001	0.18	0.11	0.107	0.35	0.11	0.002
DT Level 1 (Low vs. Mid)	0.47	0.05	< 0.001	0.40	0.05	< 0.001	0.63	0.08	< 0.001
DT Level 2 (Mid vs. High)	0.17	0.05	< 0.001	0.30	0.05	< 0.001	0.39	0.08	< 0.001
Female × Level 1	—	—	—	—	—	—	−0.46	0.10	< 0.001
Both × Level 1	—	—	—	—	—	—	−0.05	0.19	0.809
Female × Level 2	—	—	—	—	—	—	−0.10	0.10	0.334
Both × Level 2	—	—	—	—	—	—	−0.60	0.19	0.002
*Random effects*									
*σ* ^2^	0.84			0.91			0.93		
*τ*_00 ID_	0.35			0.32			0.34		
*τ*_00 face_	0.00			0.01			0.00		

*Note: N*_ID_ = 794; Observations = 2382. For narcissism and Machiavellianism the models that provided the best fit did not include interactions, hence the “—.”

The results did not support Hypothesis 1. For both Narcissism and Machiavellianism, the interaction model was not the best fitting model. For Psychopathy, the interaction effect was the opposite of what was predicted, with the high‐level DT trait reported to be the least attractive, and the differences in level being significantly greater for those with male preference.

The mean attractiveness (i.e., 1–5) ratings for STR for those with a male sexual preference (i.e., mostly women) ranged from 1.31 to 2.34 (*SD* range: 0.82–1.29; see Table S3). The mean attractiveness ratings of those with a female sexual preference (i.e., mostly men) ranged from 2.29 to 2.87 (*SD* range: 1.12–1.26).

#### 5.3.2 Long‐Term Attractiveness

We observe a similar trend for LTRs. When looking at Narcissism, those with a female preference (*β* = 0.35, *SE* = 0.05, *p* < 0.001) showed significantly higher mean attractiveness ratings than those with a male preference. Additionally, the low‐level narcissist was perceived as more attractive than the medium‐level (*β* = 1.14, *SE* = 0.05, *p* < 0.001), while the medium‐level was perceived as more attractive than the high‐level (*β* = 0.55, *SE* = 0.05, *p* < 0.001).

We observe a similar pattern once again for Machiavellianism, as those with a female preference (*β* = 0.41, *SE* = 0.07, *p* < 0.001) demonstrated significantly higher attractiveness mean ratings than those with a male preference. The low‐level was perceived as more attractive than the medium‐level (*β* = 0.98, *SE* = 0.05, *p* < 0.001), while the medium‐level was perceived as more attractive than the high‐level (*β* = 0.62, *SE* = 0.05, *p* < 0.001).

Once again, for Psychopathy, we observed those with a female preference (*β* = 0.44, *SE* = 0.05, *p* < 0.001) to show a significantly higher mean attractiveness ratings than those with a male preference (Table 11). In addition, the low‐level was perceived as more attractive than the medium‐level (*β* = 1.18, *SE* = 0.05, *p* < 0.001), while the medium‐level was perceived as more attractive than the high‐level (*β* = 0.50, *SE* = 0.05, *p* < 0.001).

**TABLE 11:** Results predicting attractiveness for LTR from DT traits (Study 2).

Predictors	Narcissism			Machiavellianism			Psychopathy		
	Estimates	*SE*	*p*	Estimates	*SE*	*p*	Estimates	*SE*	*p*
Intercept	1.87	0.04	< 0.001	1.79	0.05	< 0.001	1.75	0.04	< 0.001
Sexual preference: Female	0.35	0.05	< 0.001	0.41	0.07	< 0.001	0.44	0.05	< 0.001
Sexual preference: Both	0.31	0.09	0.001	0.31	0.09	0.001	0.32	0.09	0.001
DT Level 1 (Low vs. Mid)	1.14	0.05	< 0.001	0.98	0.05	< 0.001	1.18	0.05	< 0.001
DT Level 2 (Mid vs. High)	0.55	0.05	< 0.001	0.62	0.05	< 0.001	0.50	0.05	< 0.001
*Random effects*									
*σ* ^2^	0.81			0.86			0.81		
*τ*_00 ID_	0.15			0.13			0.16		
*τ*_00 face_	0.00			0.01			0.00		

*Note: N*_ID_ = 794; Observations = 2382.

For all three traits and for both those with a male and those with a female preference, the low‐level was the most attractive—supporting Hypothesis 2. The mean attractiveness ratings for LTR for those with a male sexual preference ranged from 1.10 to 2.80 (*SD* range: 0.39–1.33; see Table S3). The mean attractiveness ratings of those with a female sexual preference (see Table S4) ranged from 1.44 to 3.12 (*SD* range: 0.68–1.30).

#### 5.3.3 Interaction Effects of Dark Triad Levels and Relationship Type

Results indicated a significant interaction between DT levels and relationship type (Table 12). Specifically, the interaction between higher level DT traits and short‐term relationships was significant, *β* = −0.68, *SE* = 0.04, *t*(13467) = −18.37, *p* < 0.001, indicating that the positive effect of medium DT traits on attractiveness was reduced in short‐term relationships. Similarly, the interaction between higher DT traits and short‐term relationships was also significant, *β* = −0.30, *SE* = 0.04, *t*(13467) = −8.12, *p* < 0.001. This suggests that while higher DT levels generally increase attractiveness, this effect is less pronounced in short‐term relationships (see Figure S5).

**TABLE 12:** Interaction between Dark Triad levels and relationship type (Study 2).

Predictors	Attractiveness		
	Estimates	*SE*	*p*
Intercept	2.02	0.05	< 0.001
Level: 1	1.09	0.03	< 0.001
Level: 2	0.59	0.03	0.005
Relationship: Short	0.20	0.02	< 0.001
Level: 1 × Relationship: Short	−0.68	0.04	< 0.001
Level: 2 × Relationship: Short	−0.30	0.04	< 0.001
*Random effects*			
	*σ* ^2^	*τ*_00 ID_	*τ*_00 face_
	0.83	0.30	0.04

*Note: N*_ID_ = 794; Observations = 14,292.

For long‐term relationships, higher DT levels generally increased attractiveness more compared to short‐term relationships, supporting Hypothesis 2 that lower DT levels are perceived as more attractive for long‐term relationships.

#### 5.3.4 Interaction Effects of Sexual Preference and Relationship Type

The results showed a significant interaction between female sexual preference and short‐term relationships, *β* = 0.32, *SE* = 0.04, *t*(13475) = 8.73, *p* < 0.001, indicating that female preference significantly increased attractiveness in short‐term relationships (Table 13). In contrast, the interaction between preference for both sexes and short‐term relationships was not significant, *β* = 0.01, *SE* = 0.07, *t*(13475) = 0.15, *p* = 0.88. Notably, for individuals with a male preference, there was no significant interaction with relationship type, suggesting that the effect of relationship type on attractiveness was stable regardless of whether the relationship was short‐term or long‐term (see Figure S6).

**TABLE 13:** Interaction between sexual preference and relationship type (Study 2).

Predictors	Attractiveness		
	Estimates	*SE*	*p*
Intercept	1.79	0.04	< 0.001
Sexual preference: Female	0.43	0.05	< 0.001
Sexual preference: Both	0.33	0.09	< 0.001
Relationship: Short	0.02	0.03	0.46
Sexual preference: Female × Relationship: Short	0.32	0.04	< 0.001
Sexual preference: Both × Relationship: short	0.01	0.07	0.88
*Random effects*			
	*σ* ^2^	*τ*_00 ID_	*τ*_00 face_
	1.11	0.27	0.00

*Note: N*_ID_ = 794; Observations = 14,292.

### 5.4 Discussion

The fact that low level DT personalities were perceived as the most attractive for LTR purposes serves as no surprise, as it supports previous work (Carter, Campbell, and Muncer 2014; Aitken, Lyons, and Jonason 2013; Jonason, Lyons, and Blanchard 2015). What is surprising, is that to our knowledge, this is the first DT hypothetical scenario study to report findings showing low‐level DT personalities being perceived as more attractive for STR purposes than higher levels. One of the reasons could be because the study randomized the composition of the vignettes—meaning every participant saw a different composition of text, description and image. Hypothesis 2 was supported, as low‐level men and women were the most desired for LTR. This is supported by one study which did not use a control group or a physical attractiveness stimulus (Rauthmann and Kolar 2013), as well as Study 1.

Like Study 1, the findings showed that for individuals with a male preference, there was no significant interaction with relationship type, suggesting that the effect of relationship type on attractiveness was stable regardless of whether the relationship was short‐term or long‐term. This does not support Hypothesis 1, as higher DT levels did not significantly increase attractiveness for those with a male preference in short‐term relationships. For Hypothesis 2, the results suggest that attractiveness ratings were more stable for male preferences across relationship types.

## 6 General Discussion

The aim of this project was to replicate and extend previous work (Rauthmann and Kolar 2013; Carter, Campbell, and Muncer 2014; Aitken, Lyons, and Jonason 2013; Dufner et al. 2013; Jonason, Lyons, and Blanchard 2015).

While the overall mean attractiveness ratings were low, Study 1 showed that individuals with a male sexual preference (mostly women) preferred medium and sometimes higher DT levels for STR on average. In Study 2, Hypothesis 1 was not supported. To our knowledge, Study 2 is the first study to demonstrate women preferring lower‐level DT personalities for STR. There are a number of reasons that could explain why the findings from Study 2 defer from previous work that assessed DT attractiveness using behavioral stimuli:

1. Sample size—all the available studies we found on the topic (Rauthmann and Kolar 2013; Carter, Campbell, and Muncer 2014; Dufner et al. 2013, Study 1; Aitken, Lyons, and Jonason 2013; Jonason, Lyons, and Blanchard 2015) consisted of samples below 150.
2. Age of participants—the majority of previous research consists of younger (usually female) students (Rauthmann and Kolar 2013, *M*_age_ = 23.78; Carter, Campbell, and Muncer 2014, *M*_age_ = 19.4; Aitken et al., Study 1, *M*_age_ = 22.75). Youthful student populations tend to be more short‐term oriented compared to a more representative samples (Carter, Campbell, and Muncer 2014). Half of the participants (50.69%) from our second study were not students. Moreover, the female participants from our second study appear to be substantially older (*M*_age_ = 29.37) than any of the populations from the discussed studies.
3. Stimuli for physical attractiveness—all of the mentioned studies used only descriptions of behavior with no faces of the hypothetical targets. The only exception was Dufner et al. (2013, Study 2), however the targets in that study were real people and they were only rated for attractiveness by one person chosen by the target. In 97.36% of cases, the rater was someone who they knew. The addition of facial images of a controlled level of attractiveness to vignettes does change the rating task asked of participants. However, we would argue that the combination of visual stimuli and descriptor is closer to real‐world situations than relying on a person's imagination of a target when reading descriptions of behavior. In our work, Study 1 consisted of descriptions of behavior that were attached to specific faces. In other words, the combination of face and behavior (e.g., high‐level narcissist + face C) was consistent across the participants. We used the same faces for Study 2, only this time the combinations were randomized, meaning every participant saw a different combination of behavior and face. This produced different results between the two studies. Although we controlled for physical attractiveness of the faces, it is likely that some faces might still have been considered more attractive than others. This could explain the difference between the studies suggesting that the concept of the DT personality is only attractive when is accompanied by higher physical attractiveness or when the attractiveness of the face is left to the participant's imagination.
4. Medium level—only one of the mentioned studies (Dufner et al. 2013, Study 1) adopted a medium‐level DT target to be assessed by participants. To our knowledge, our work is the second study to provide a medium‐level—a variable which likely influences the overall findings.
5. Sex of the assessed targets—all of the discussed studies used hypothetical targets that were either entirely male or were based on the opposite sex of the participant. To our knowledge, our work is the first to use targets based on the sexual orientation of participants.

The contrasting results between Studies 1 and 2 presented here, and the extant literature, likely result from a combination of the factors discussed above. However, the general lack of consensus with the previous literature, when considered alongside the developments in methodology used in the current study, would indicate that the supposed attractiveness of DT individuals for specific types of relationships, is more complex than previously thought. It is likely that any potential attraction to DT individuals only occurs when physical attractiveness is present.

In summary, we see the results presented here as posing a number of questions for the field: Can we realistically measure DT attractiveness if we are not including a physical component as a stimulus? If we do in fact include a physical component, are we then measuring DT attractiveness or are we mostly extracting the perception of the face? How our findings are interpreted will likely be determined by how the reader responds to these questions. It could be that there is no reliable way to realistically measure this concept without introducing confounding variables. Either way, we believe more caution and less sensationalism regarding the concept of DT attractiveness will certainly benefit psychological research.

### 6.1 Limitations and Future Directions

Considering the experimental nature of this study, it is important to interpret these findings with caution. First, there was no second pilot study ran to assess and compare the refined vignettes following the feedback from subject matter experts. Second, while the vignettes were modified and changed, the base of the vignettes still originated from the Dirty Dozen (Jonason and Webster 2010), a short measurement that has received substantial criticism for its construct validity when distinguishing Machiavellianism and psychopathy (Miller et al. 2012). Third, participants were not asked whether they are currently in a relationship. This is a limitation, as previous work has suggested relationship status influences women's perception on male faces (Little et al. 2007).

## 7 Conclusion

The current findings do not provide support for the notion that Dark Triad (DT) traits are attractive. The results suggest that men tend to be less selective than women overall when it comes to choosing partners—this occurs for both short‐term and long‐term relationships. The rank‐order gender differences from Study 1 appear to disappear for both STR and LTR when the composition of the vignettes was randomized in Study 2. The faces used in Studies 1 and 2 were mostly of average attractiveness. Given that previous evidence supports the idea that DT is attractive, it may be that DT interacts with attractiveness, while DT increases the attractiveness of faces that are already considered attractive.

## Author Contributions

Yavor Dragostinov and Tom Booth contributed equally to this study.

## Conflicts of Interest

The authors declare no conflicts of interest.

## Supporting information


Data S1.

## Data Availability

The code, data and study materials that may be used to replicate all the analyses can be found here https://osf.io/fzc37/.

## Endnotes

^1^
Please note that this is the mean age of only 266 of the participants. Due to an error in the survey, more than half the participants were only asked to confirm if they were at least 18 years old rather than what age they are.
