# Supplementary material for: The (Un)Attractiveness of Dark Triad Personalities: Assessing Fictitious Characters for Short‐ and Long‐Term Relationships
Source: J Pers. 2024 Nov 18;93(5):1097–111. doi: 10.1111/jopy.12994 (PMC12421710; doi:10.1111/jopy.12994)
Supplement: Supplementary file 1 — Data S1. [file JOPY-93-1097-s001.docx]

**ONLINE SUPPLEMENTARY MATERIAL**

**The (Un)Attractiveness of Dark Triad Personalities: Assessing Fictitious Characters for Short- and Long-Term Relationships**

**Yavor Dragostinov^*1,2^ & Tom Booth^1^**

^*^The corresponding author’s current affiliation is the University of Texas at Austin: y.dragostinov@sms.ed.ac.uk

yavor.dragostinov@austin.utexas.edu

^1^ University of Edinburgh, Department of Psychology, Edinburgh UK

^2^ Heriot-Watt University, School of Social Sciences, Edinburgh UK

**Vignette Development**

*The list of the original vignettes we used:*

**Narcissism**

**HIGH**

Jake’s colleagues describe him as someone who always wants others to pay

attention to him. He often expects favors from others and is frequently seeking

prestige or status. Even Jake describes himself as egocentric and also feels that he

is better than everyone else.

**LOW**

Harry expects the same treatment from everyone at work and doesn’t perceive

himself as more special than others. During conversations, he tends to listen and

sympathize and doesn’t feel the need to dominate social situations. Harry has a low

sense of self-importance.

**MEDIUM**

Daniel sometimes wants others to admire him and what he does, but also finds that

he is not bothered if people pay attention to him. He also does not really seek kudos

from others but he has been known to expect special favours from them from time to

time.

**Machiavellianism**

**HIGH**

David has trouble understanding people’s feelings. He has a reputation of using

deceit and lying in order to get his way. He tends to use flattery so he can reach his

aims. Often, David would exploit other via manipulation towards his own end.

**LOW**

Russell is not known for engaging in lying and deceitful behaviors. He does not

manipulate others and appears to have no trouble understanding people’s feelings.

Russell is very honest and does not use flattery in order to achieve his ways.

**MEDIUM**

Rob admits he has lied on occasion to get his own way, but avoids manipulating

others to achieve this. He has sometimes tried to exploit others for his own end but

never flatters other people in doing so.

**Psychopathy**

**HIGH**

Josh has a reputation of getting frustrated easily. He is not too concerned with the

morality of his actions and both clients and subordinates have described him as

lacking remorse and being insensitive.

**LOW**

Mike is a calm individual who does not lose his temper quickly. He does not engage

in cynical comments and is perceived as a remorseful and sensitive person.

**MEDIUM**

Paul can tend to be callous and insensitive with other people, but he does express

remorse when he has done. He is not particularly cynical of other people but he

admits he is not always concerned with the morality of his actions.

*The list of the updated vignettes following consulting with subject matter experts.*

**Narcissism**

**HIGH**

Jake is described as someone who always wants others to pay attention to him. He often expects favours from others and is frequently seeking prestige or status. Even Jake describes himself as egocentric.

**LOW**

Harry expects the same treatment from everyone and doesn’t perceive himself as more special than others. During conversations, he tends to listen and sympathise.

**MEDIUM**

Daniel occasionally wants others to admire him and what he does. He does not really seek attention from others but he has been known to expect special favours from them.

**Machiavellianism**

**HIGH**

David does not think people will work hard unless they are forced to. He has a reputation of using deceit and lying in order to get his way. Often, David would exploit others via manipulation towards his own end.

**LOW**

Russell doesn’t believe it is wise to flatter important people. He does not manipulate others and always tells the truth.

**MEDIUM**

Rob admits he has used manipulation at times, but he doesn’t engage in it regularly. Although he frequently questions people’s motives, he doesn’t believe everyone is vicious.

**Psychopathy**

**HIGH**

Josh has a reputation of getting angry easily. He is not at all concerned with the morality of his actions and people described him as lacking remorse.

**LOW**

Mike is a calm individual who does not lose his temper quickly. He is perceived as a remorseful and sensitive person.

**MEDIUM**

Paul can get frustrated fairly easily. He admits he is not always concerned with the morality of his actions, although he does feel regret after crossing the line.

**Mean Averages of Attractiveness**

**Table S1.**

Mean and standard deviation from participants with a male sexual preference (Study 1).

| Trait | Relationship Type | Mean | SD |
| --- | --- | --- | --- |
| Narcissism | | | |
| Low | Short | 2.05 | 1.02 |
|  | Long | 2.37 | 1.14 |
|  | | | |
| Medium | Short | 1.98 | 1.05 |
|  | Long | 1.74 | 0.93 |
|  | | | |
| High | Short | 2.09 | 1.08 |
|  | Long | 1.37 | 0.70 |
|  | | | |
| Machiavellianism | | | |
| Low | Short | 1.88 | 1.08 |
|  | Long | 2.16 | 1.14 |
|  | | | |
| Medium | Short | 2.19 | 1.13 |
|  | Long | 2.06 | 1.08 |
|  | | | |
| High | Short | 1.55 | 0.85 |
|  | Long | 1.14 | 0.51 |
|  | | | |
| Psychopathy | | | |
| Low | Short | 1.82 | 1.03 |
|  | Long | 2.34 | 1.20 |
|  | | | |
| Medium | Short | 2.01 | 0.97 |
|  | Long | 1.69 | 0.84 |
|  | | | |
| High | Short | 1.39 | 0.70 |
|  | Long | 1.12 | 0.39 |

**Table S2.**

Mean and standard deviation scores on participants with a female sexual preference (Study 1).

| Trait | Relationship Type | Mean | SD |
| --- | --- | --- | --- |
| Narcissism | | | |
| Low | Short | 3.18 | 1.14 |
|  | Long | 3.37 | 1.12 |
|  | | | |
| Medium | Short | 2.50 | 1.23 |
|  | Long | 1.99 | 1.06 |
|  | | | |
| High | Short | 2.42 | 1.24 |
|  | Long | 1.52 | 0.91 |
|  | | | |
| Machiavellianism | | | |
| Low | Short | 1.99 | 1.13 |
|  | Long | 2.38 | 1.26 |
|  | | | |
| Medium | Short | 2.00 | 1.05 |
|  | Long | 1.78 | 0.95 |
|  | | | |
| High | Short | 2.01 | 1.11 |
|  | Long | 1.35 | 0.83 |
|  | | | |
| Psychopathy | | | |
| Low | Short | 3.10 | 1.25 |
|  | Long | 3.39 | 1.33 |
|  | | | |
| Medium | Short | 2.46 | 1.18 |
|  | Long | 1.99 | 1.09 |
|  | | | |
| High | Short | 2.20 | 1.26 |
|  | Long | 1.33 | 0.74 |

**Table S3.**

Mean and standard deviation from individuals with a male sexual preference (Study 2).

| Trait | Relationship Type | Mean | SD |
| --- | --- | --- | --- |
| Narcissism | | | |
| Low | Short | 2.34 | 1.18 |
|  | Long | 2.80 | 1.27 |
|  | | | |
| Medium | Short | 1.73 | 0.96 |
|  | Long | 1.64 | 0.91 |
|  | | | |
| High | Short | 1.49 | 0.82 |
|  | Long | 1.16 | 0.53 |
|  | | | |
| Machiavellianism | | | |
| Low | Short | 2.31 | 1.18 |
|  | Long | 2.65 | 1.25 |
|  | | | |
| Medium | Short | 1.81 | 1.03 |
|  | Long | 1.65 | 0.92 |
|  | | | |
| High | Short | 1.43 | 0.84 |
|  | Long | 1.11 | 0.40 |
|  | | | |
| Psychopathy | | | |
| Low | Short | 2.33 | 1.29 |
|  | Long | 2.74 | 1.33 |
|  | | | |
| Medium | Short | 1.70 | 0.93 |
|  | Long | 1.42 | 0.68 |
|  | | | |
| High | Short | 1.31 | 0.67 |
|  | Long | 1.10 | 0.39 |

**Table S4.**

Mean and standard deviation from participants with a female sexual preference (Study 2).

| Trait | Relationship Type | Mean | SD |
| --- | --- | --- | --- |
| Narcissism | | | |
| Low | Short | 2.87 | 1.16 |
|  | Long | 3.12 | 1.22 |
|  | | | |
| Medium | Short | 2.52 | 1.15 |
|  | Long | 2.07 | 1.00 |
|  | | | |
| High | Short | 2.34 | 1.13 |
|  | Long | 1.45 | 0.70 |
|  | | | |
| Machiavellianism | | | |
| Low | Short | 2.84 | 1.20 |
|  | Long | 3.06 | 1.25 |
|  | | | |
| Medium | Short | 2.50 | 1.15 |
|  | Long | 2.08 | 1.01 |
|  | | | |
| High | Short | 2.29 | 1.12 |
|  | Long | 1.44 | 0.72 |
|  | | | |
| Psychopathy | | | |
| Low | Short | 2.74 | 1.26 |
|  | Long | 3.08 | 1.30 |
|  | | | |
| Medium | Short | 2.57 | 1.13 |
|  | Long | 2.07 | 0.93 |
|  | | | |
| High | Short | 2.27 | 1.17 |
|  | Long | 1.44 | 0.68 |

**Sensitivity Analysis**

We ran every model twice – once with all of the participants and one with only the heterosexual participants. Code for generating the plots can be found at <https://osf.io/fzc37/>.

**Figure S1.**

**Model comparison for Study 1.**

**Figure S2. Model comparison for Study 2**.

**Figure S3.
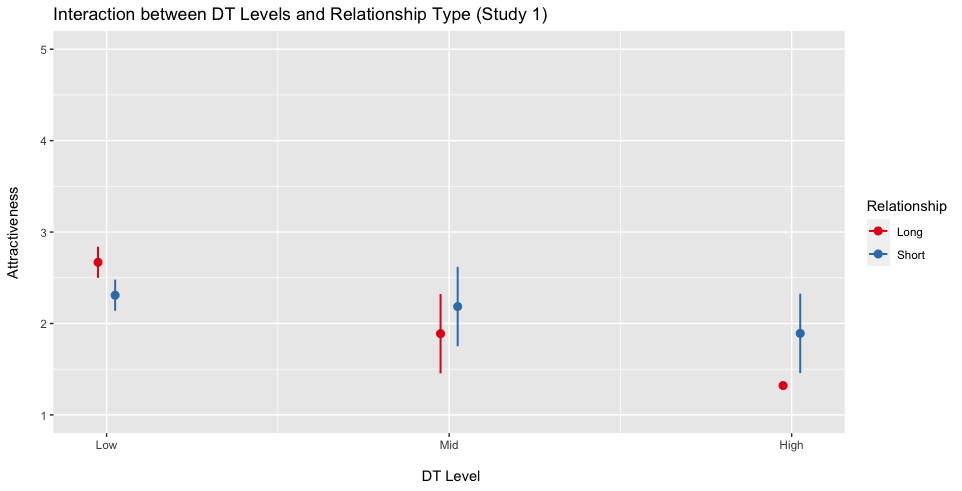
**

**Figure S4.**

**
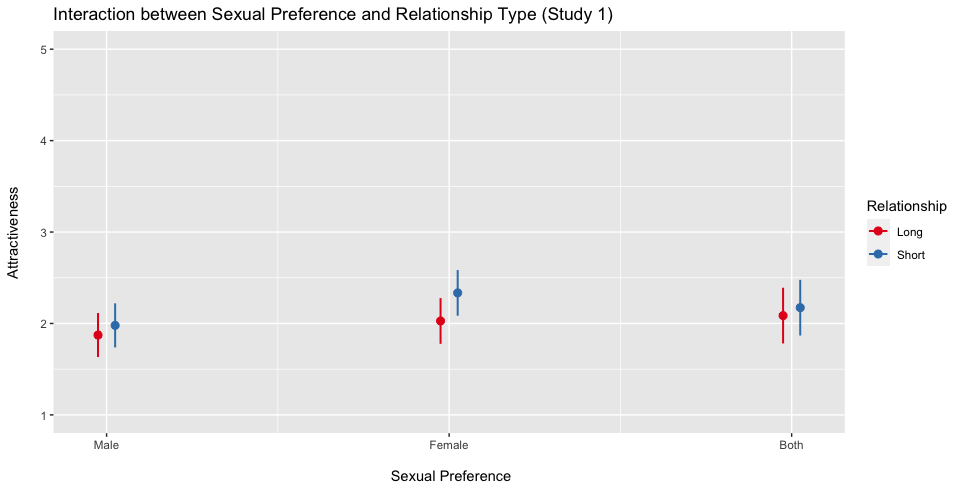
**

**Figure S5.**

**
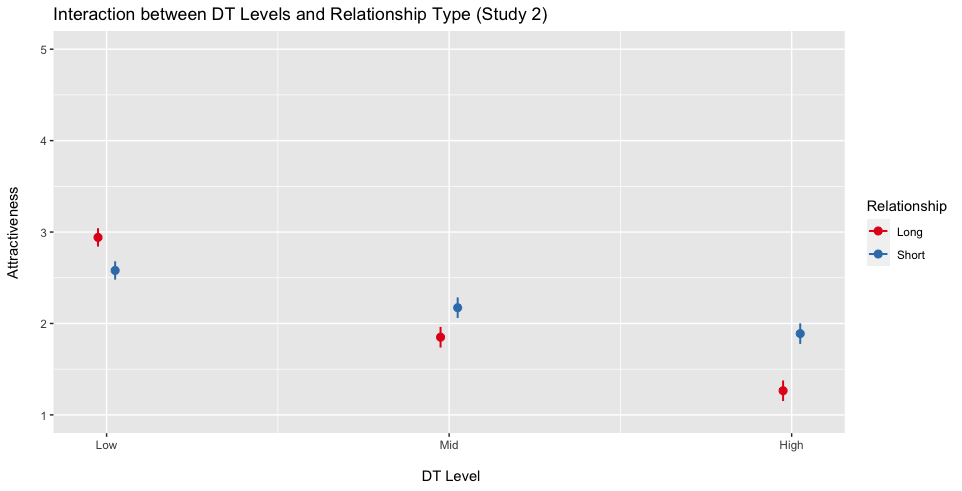
**

**Figure S6.**

**
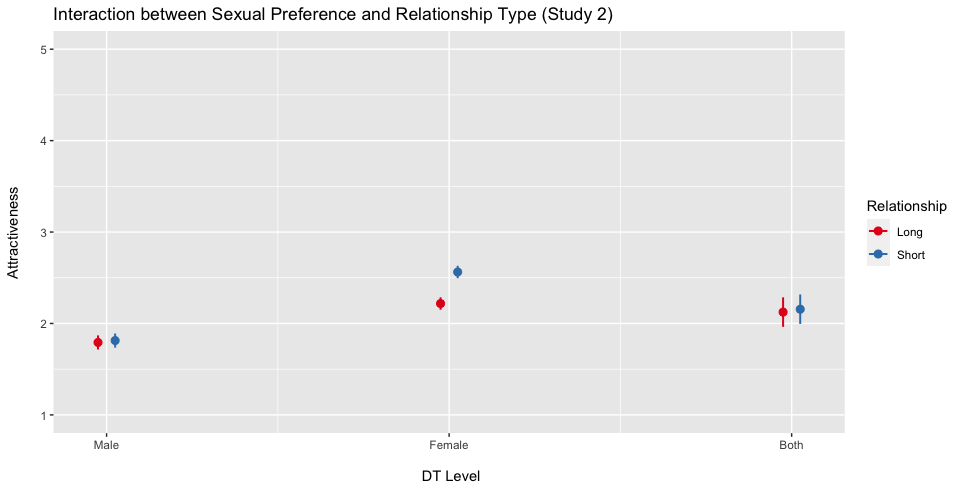
**
